# Fecal Host Transcriptomics for Non-invasive Human Mucosal Immune Profiling: Proof of Concept in *Clostridium difficile* Infection

**DOI:** 10.20411/pai.v3i2.250

**Published:** 2018-09-12

**Authors:** Robert Schlaberg, Amanda Barrett, Kornelia Edes, Michael Graves, Litty Paul, Jenna Rychert, Bert K. Lopansri, Daniel T. Leung

**Affiliations:** 1 Department of Pathology, University of Utah School of Medicine, Salt Lake City, Utah; 2 ARUP Laboratories, Salt Lake City, Utah; 3 Division of Infectious Diseases, University of Utah School of Medicine, Salt Lake City, Utah; 4 Division of Infectious Diseases and Clinical Epidemiology, Intermountain Medical Center, Murray, Utah

**Keywords:** Clostridium difficile, host transcriptomics, fecal transcriptomics, actin-cytoskeleton, mucosal immune response

## Abstract

**Background:**

Host factors play an important role in pathogenesis and disease outcome in *Clostridium difficile* infection (CDI), and characterization of these responses could uncover potential host biomarkers to complement existing microbe-based diagnostics.

**Methods:**

We extracted RNA from fecal samples of patients with CDI and profiled human mRNA using amplicon-based next-generation sequencing (NGS). We compared the fecal host mRNA transcript expression profiles of patients with CDI to controls with non-CDI diarrhea.

**Results:**

We found that the ratio of human actin gamma 1 (ACTG1) to 16S ribosomal RNA (rRNA) was highly correlated with NGS quality as measured by percentage of reads on target. Patients with CDI could be differentiated from those with non-CDI diarrhea based on their fecal mRNA expression profiles using principal component analysis. Among the most differentially expressed genes were ones related to immune response (IL23A, IL34) and actin-cytoskeleton function (TNNT1, MYL4, SMTN, MYBPC3, all adjusted *P*-values < 1 x 10^-3^).

**Conclusions:**

In this proof-of-concept study, we used host fecal transcriptomics for non-invasive profiling of the mucosal immune response in CDI. We identified differentially expressed genes with biological plausibility based on animal and cell culture models. This demonstrates the potential of fecal transcriptomics to uncover host-based biomarkers for enteric infections.

## INTRODUCTION

*Clostridium* (or *Clostridioides*) *difficile* is a spore-forming, toxin-producing bacterium that causes diarrhea and colitis, most often associated with antibiotic use. It is the most common hospital-associated infection in the United States, with an estimated 453,000 cases and more than 29,000 deaths annually [1]. While *C. difficile* infection (CDI) is most often acquired in healthcare settings, community-associated infections are increasing [2, 3]. The most commonly used diagnostics for CDI are based on detection of the toxin or toxin genes. Due to their high sensitivity and rapid turnaround times, nucleic acid amplification tests (NAATs) have been widely adopted for the diagnosis of CDI. However, NAAT-based detection of *C. difficile* genomic DNA is unable to differentiate true CDI from colonization in the presence of alternative causes of diarrhea. Adoption of NAATs has led to an increase in CDI diagnoses worldwide, and concerns of over-diagnosis of CDI resulting in unnecessary use of antibiotics have sparked renewed interest in improved diagnostic strategies [4, 5].

Host biomarkers have the potential to complement microbe-based tests in the diagnostic algorithm. Unfortunately, currently available fecal biomarkers, including lactoferrin and calprotectin, have on the whole been unable to differentiate CDI from other causes of diarrhea [6, 7]. Furthermore, host factors play an important role in pathogenesis and disease outcome in CDI [8], and characterization of the host response could uncover novel biomarkers. Animal models have demonstrated that mice deficient for NOD1 [9], MyD88 [10], TLR4 [11], and innate lymphoid cells [12] are more susceptible to CDI, and that mice lacking IL-23 are more protected from CDI [13]. However, these findings have not been thoroughly tested in human studies. Limited hypothesis-driven studies in humans have identified several host factors associated with disease, such as lower levels of antibody against *C. difficile* toxin A [14, 15], IL-8 polymorphism [16], and elevated fecal cytokines [17, 18].

Attempts at investigating intestinal immune responses in human CDI have been limited by the ethical and logistical challenges of invasive procedures. However, recent technological advances have enabled the noninvasive profiling of the mucosal immune responses by host transcriptomic profiling of fecal specimens using RNA-seq, microarrays, and PCR [19–21]. Thus, the objective of this study was to develop and optimize an unbiased method for examining the fecal host transcriptome in patients with *C. difficile* infection.

## METHODS

### Patient Selection

We collected de-identified fecal samples from adult patients whose feces were sent to the Inter-mountain Healthcare microbiology laboratory for *C. difficile* testing. Patients were defined to have CDI by positive results for GDH and toxin enzyme immunoassay (C. diff Quik Chek Complete, Alere). Controls were randomly selected from diarrheal samples sent for *C. difficile* testing but had negative GDH and toxin enzyme immunoassay results. Samples were de-identified and only data regarding age, gender, CDI status (positive or negative), and CDI severity were linked to the stools by a study number. Severe disease was determined by chart review using the modified University of Illinois criteria excluding the presence of pseudomembranous colitis [22, 23]. The study protocol was submitted to the Institutional Review Boards of the University of Utah and Intermountain Healthcare and deemed to be exempt from review.

### Sample Collection and Preservation

The primary stool specimens were kept refrigerated after testing. After a positive toxin result, an aliquot of up to 4 mL of residual feces, not used for microbiologic testing, was placed into 4 mL of RNAprotect Cell Reagent (Qiagen) and stored at -80°C until RNA extraction.

### RNA Extraction

Fecal samples were thawed, vortexed, and filtered through gauze with addition of 5-10 mL of PBS. The filtrate was pelleted by centrifugation with the gauze still in the tube for 5 minutes at 500*g*; then the gauze was discarded, the sample vortexed to resuspend the pellet, and the suspension was filtered through a 40 μm cell strainer (Fisherbrand). The resulting filtrate was centrifuged again at 500*g*, and the supernatant discarded. The remaining pellet was mixed with lysis buffer (PureLink RNA Mini Kit, LifeTechnologies), and cells were lysed by vortexing for 2 minutes in tubes containing 0.5 mm glass beads (PowerBead Tubes, MoBio). Equal volumes of 70% ethanol were added, the mix centrifuged, and the supernatant transferred to a PureLink spin column. The remainder of the RNA isolation steps was completed according to the PureLink RNA Mini Kit manufacturer's instructions, including use of on-column PureLink DNase treatment.

### Quantitative RT-PCR

RNA was quantified by Qubit and Nanodrop 1000 (both Thermo Fisher Scientific). An aliquot of 8 μL of extracted RNA was reverse transcribed with SuperScript IV and VILO master mix (Invitrogen) primed with random hexamers. To assess integrity and yield of human mRNA, transcripts for a human housekeeping gene (actin gamma 1, ACTG1, Hs03044422_g1) and bacterial 16S rRNA (16S, Ba04230899_s1, both Thermo Fisher Scientific) were quantified by quantitative reverse transcriptase PCR (RT-qPCR), TaqMan Fast Advanced Master Mix (Thermo Fisher Scientific), and a QuantStudio 3 real-time thermocycler (Thermo Fisher Scientific). Amplification conditions were 50°C for 2 minutes, 95°C for 20 seconds, followed by 40 cycles of 95°C for 1 second and 60°C for 20 seconds. Commercial human RNA (Human Control RNA, Thermo Fisher Scientific) and total RNA extracted from a pure culture of Escherichia coli (DH5α) were used as standards. A ratio of ACTG1 to 16S was calculated and compared to quality transcriptomic data.

### Library Preparation for Transcriptome Sequencing

Libraries were prepared with the Ion AmpliSeq Transcriptome Human Gene Expression kit (Thermo Fisher Scientific, version 1) using 10 or 100 ng of total RNA, depending on human mRNA abundance. Commercial human RNA was used as control. Targets were amplified with the Ion AmpliSeq Transcriptome Human Gene Expression Core Panel targeting > 20,000 genes, followed by partial digestion of primers, ligation of barcoded adapters, and library amplification for 18 cycles. Amplified libraries were eluted in 30 uL of low TE buffer after purification and quantified using the Ion Library TaqMan Quantitation Kit (Thermo Fisher Scientific). Libraries from 12 samples with ACTG1:16S ratios of above 10^-4^ were randomly selected for sequencing on the Ion Torrent Proton system using a P1 chip according to the manufacturer's instructions (Thermo Fisher Scientific).

### Analysis of Transcriptome Sequencing Data

The resulting sequencing data was analyzed using the Torrent Suite (Thermo Fisher Scientific, version 5.0.4 and the human reference genome build hg19) with default analysis parameters providing sequencing read counts for each of the targeted genes. The resulting read count matrices were analyzed using DESeq2 [24] within R (R Foundation for Statistical Computing, Vienna, Austria; version 3.3.2). Genes with *P*-values adjusted for multiple testing (Benjamini and Hochberg, generated within DESeq2) < 0.05 were included in subsequent analyses. For quality control and to assess adequacy of calculated percentage of reads on target, these resulting sequencing reads were also analyzed with Taxonomer [25], and reads binned as human were quantified and compared to percentage of reads on target provided by the Torrent Suite software. Functional annotations were derived using DAVID [26] analyses using Benjamini-adjusted *P*-values.

## RESULTS

### Human Feces mRNA Yield and Effect on Transcriptome Profiling

**Figure 1.**
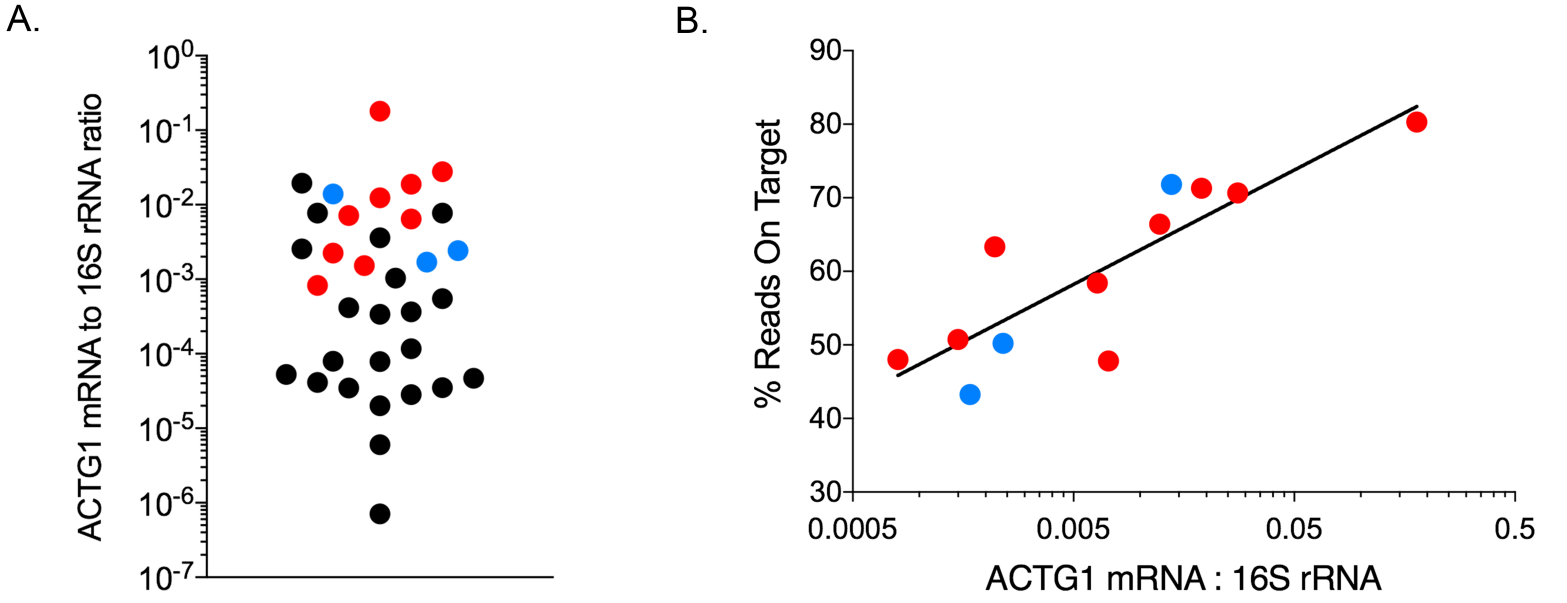
**Quality control of extracted RNA and sequencing Data.** A) ACTG1 mRNA to 16S rRNA ratios for 34 fecal samples; samples highlighted in red (CDI) and blue (diarrhea of other causes) were selected for sequencing. B) ACTG1:16S ratios correlated with expected sequences (*reads on target*, Pearson R^2^ 0.396; Goodness of Fit (semilog) R^2^ = 0.73).

**Supplementary Figure 1.**
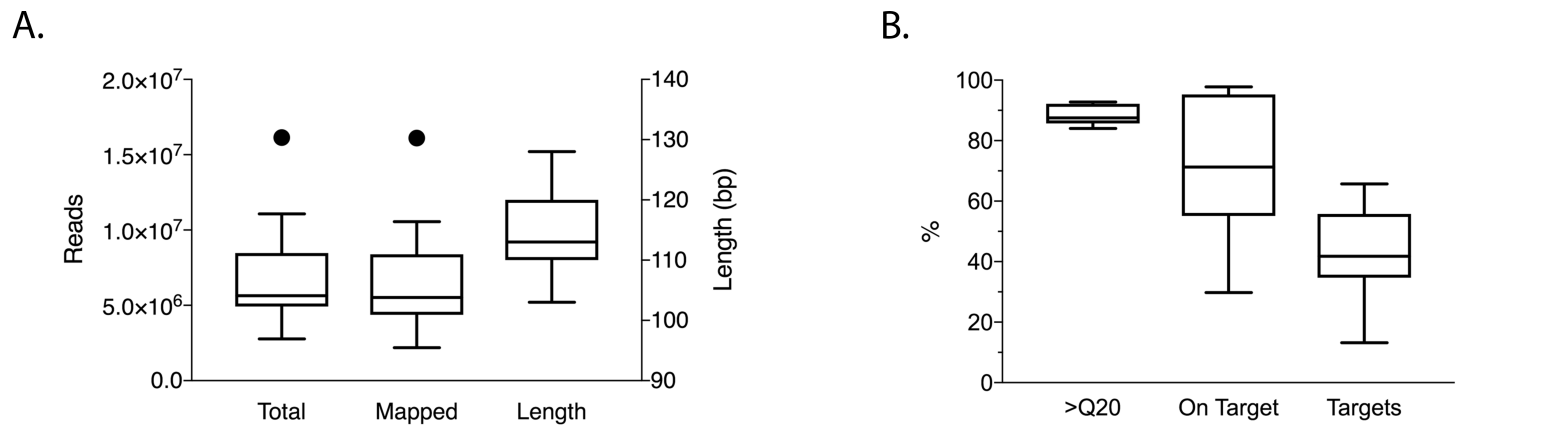
Sequencing quality metrics for expression profiling of mRNA extracted from feces of patients with CDI and diarrhea of other causes.

**Supplementary Figure 2.**
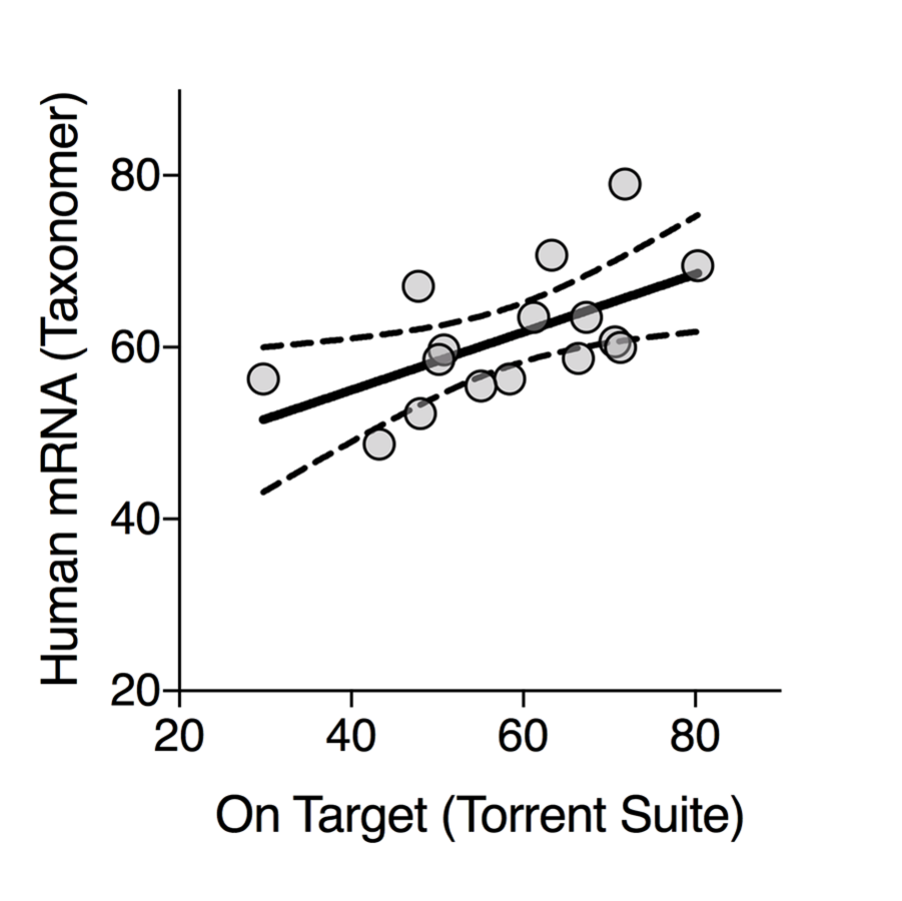
Correlation of sample composition and expression profiling by Taxonomer (y-axis, percentage of human mRNA reads) and Torrent Suite (x-axis, percentage reads on target).

The AmpliSeq Transcriptome Human Gene Expression kit is intended for analysis of 10 ng of human RNA. To assess limitations of analyzing mixed bacterial and human RNA from fecal samples, we quantified the proportion of human ACTG1 mRNA and bacterial 16S RNA in 34 fecal samples by qPCR. Estimated ACTG1 mRNA and 16S rRNA (ACTG1:16S) ratios were calculated to assess human mRNA concentrations. ACTG1:16S ratios spanned 5 orders of magnitude (Figure 1A). RNA from fecal specimens with ACTG1:16S ratios above the median (6.1 x 10^-4^) were randomly selected for transcriptome analysis (Figure 1A, highlighted in red and blue). A mean of 4.7 x 10^6^ reads (SD = 1.9 x 10^6^) were generated per sample with a mean 86.0% of base calls having quality scores of ≥ Q20 (SD 1.5%). On average, 58.5% of reads contained expected sequences or *reads on target* (ie, sequencing reads aligning to the expected regions of the > 20,000 targeted genes, SD 13.1%) and 34.5% of targeted genes were detected (SD 9.2%, Supplementary Figure 1). To assess accuracy of data analysis with the Torrent Suite when applying the method to mixed RNA with a high abundance of non-human RNA, we also analyzed the sequencing reads with a metagenomics data analysis tool able to enumerate human mRNA reads in highly-mixed data (Taxonomer) [25]. The proportion of human mRNA reads as determined by Taxonomer was highly correlated with the percentage of reads on target as determined by the Torrent Suite (Supplementary Figure 2). Subsequent analyses are based on results of the Torrent Suite. The ACTG1:16S ratio correlated with the percentage of valid reads (R^2^ = 0.73, Figure 1B) supporting its use as a predictor for sample suitability for transcriptome analysis.

### Transcriptome Profiling in C. difficile-Positive and C. difficile-Negative Fecal Samples

**Figure 2.**
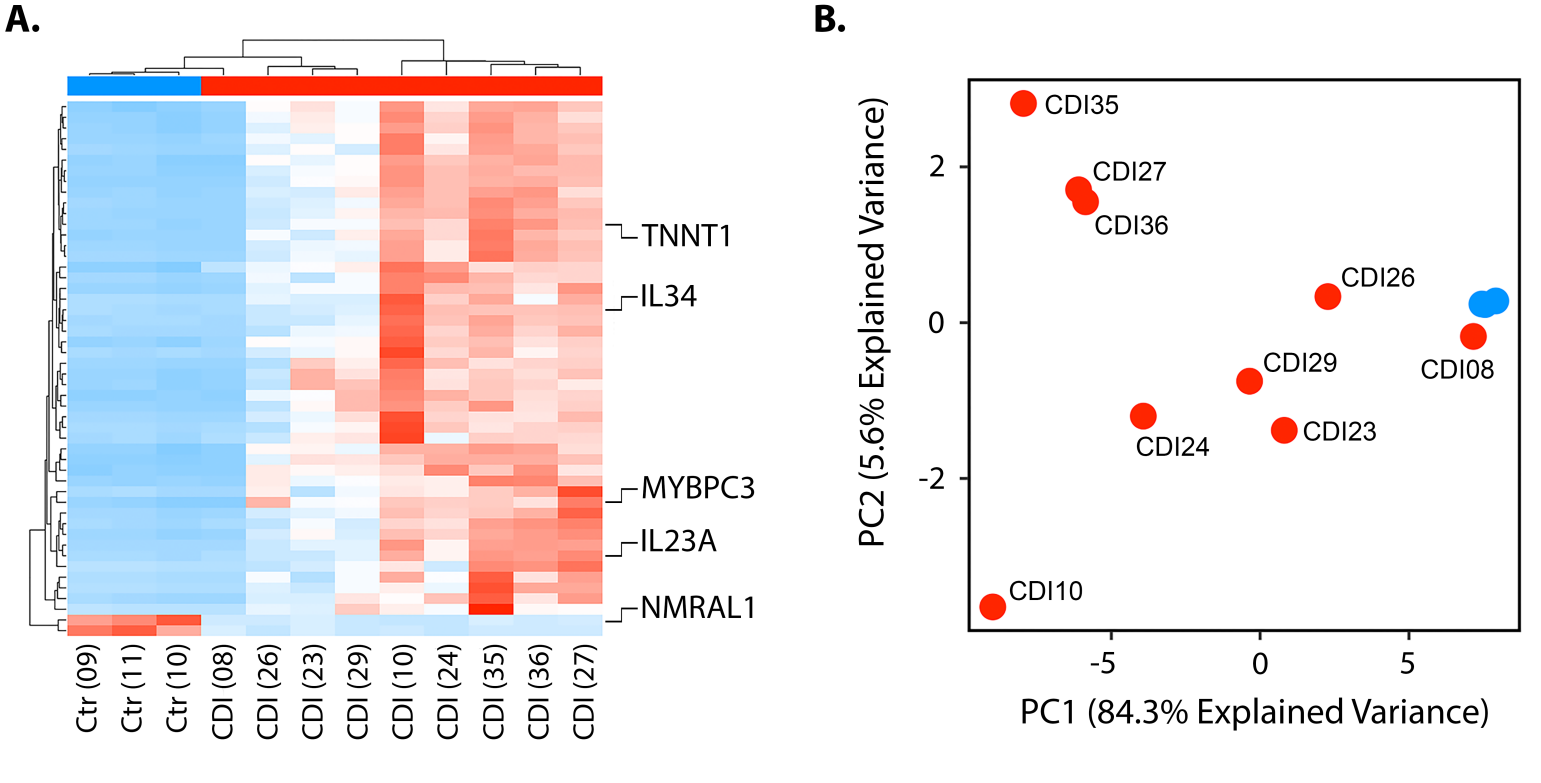
**mRNA expression in patients with CDI vs controls.** A) Relative expression levels of the top 50 of these differentially expressed genes between patients with CDI and diarrhea of other causes. B) Principal component analysis demonstrated 84.3% of the variance explained by principal component (PC) 1.

Of the 12 samples sequenced, 9 (75%) were from patients with CDI and 3 (25%) were from patients with diarrhea of other causes (Table 1). Of the 9 samples from CDI patients, the median age was 62 years (range 17 - 91), 5 were female, 5 had recurrent episodes, and none met Zar criteria for severe CDI [23]. A total of 20,046 genes (96.3%) had ≥ 1 read in at least 1 sample, with 6388 genes (31.9%) being differentially expressed between patients with CDI and diarrhea of other causes based on an adjusted *P*-value of < 0.05 (DESeq2). To identify genes that were consistently and maximally differentially expressed, we limited subsequent analyses to genes with < 80% coefficient of variation within each of the 2 groups and > 10-fold difference between group means (n = 922). Relative expression levels of the top 50 of these differentially expressed genes are shown in Figure 2A. Among these genes were several with a known role in the pathogenesis of CDI, including IL23A (encoding for the alpha subunit of interleukin 23), IL34 (interleukin 34), TNNT1 (slow skeletal troponin type 1), MYBPC3 (myosin-binding protein C isoform 3), and NMRAL1 (NmrA like redox sensor 1). Of the 9 patients with CDI, 1 showed an mRNA expression profile that was more similar to those of the controls (CDI08), 3 had intermediary mRNA expression profiles (CDI23, CDI26, CDI29), while the remaining 5 (CDI10, CDI24, CDI27, CDI35, CDI36) were characterized by high expression levels of most of the differentially expressed genes (Figure 2A). In a principal component analysis of these expression profiles > 84% of the variance was explained by principal component (PC) 1, which also separated the 3 groups outlined above (Figure 2B). There were no clear differences between groups in age, gender, or recurrence status.

**Table 1:** Demographic and clinical data for patients with CDI and controls with non-CDI diarrhea

Sample ID	Group	Recurrent Episode	Age	Gender
08	CDI	Yes	73	M
10	CDI	No	70	M
23	CDI	Yes	62	M
24	CDI	No	17	M
26	CDI	Yes	46	F
27	CDI	Yes	80	F
29	CDI	No	80	F
35	CDI	Yes	31	F
36	CDI	No	19	F
N9	Control	-	30	F
N10	Control	-	52	F
N11	Control	-	64	M

**Figure 3.**
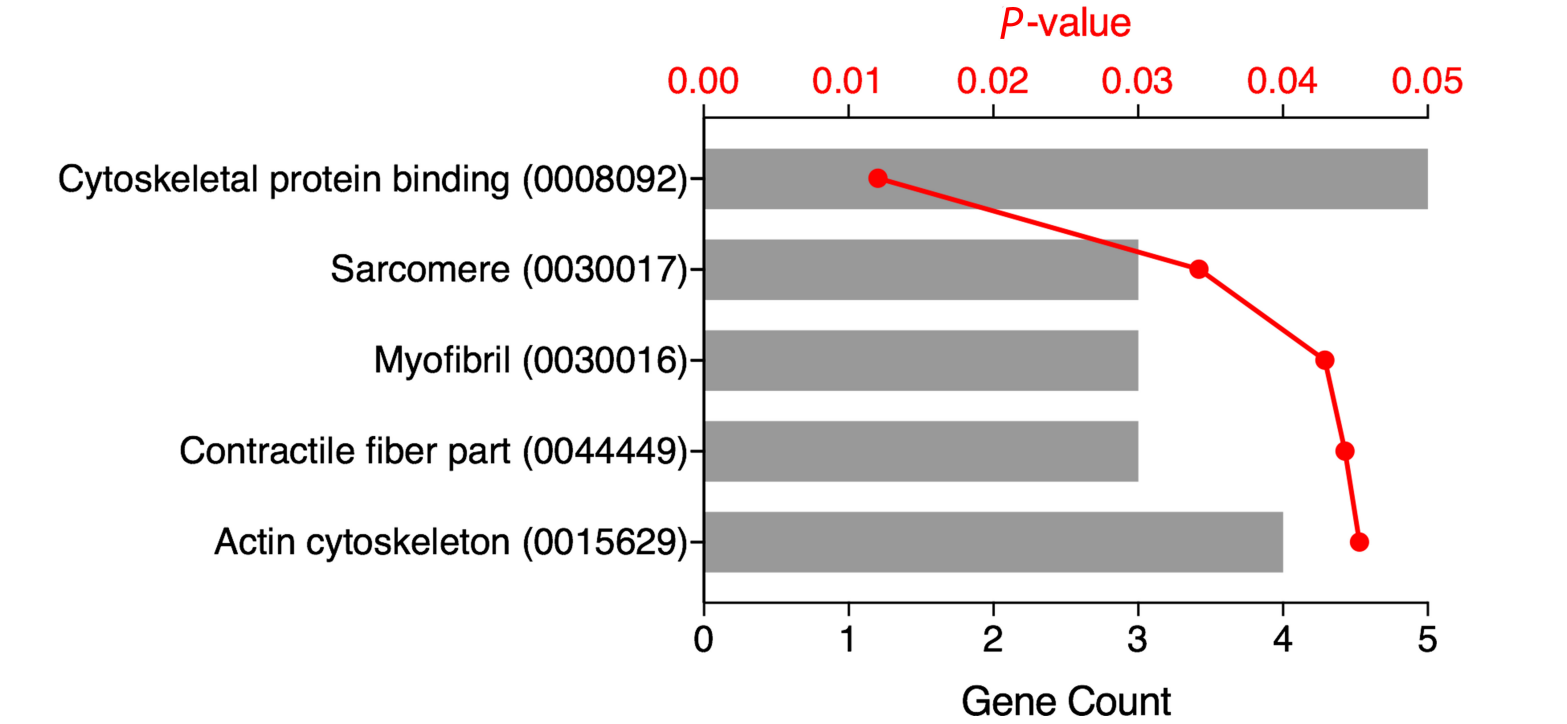
**Functional Annotation.** Gene Ontology (GO) terms and accession numbers (in parenthesis) for the top 50 differentially expressed genes (DAVID analysis, GO terms with Benjamini-adjusted *P*-values < 0.05) are shown with the number of genes (grey bar) and adjusted *P*-value (red).

DAVID analysis of the top 50 differentially expressed genes (Supplementary Table 1) showed 5 functional annotations to be significantly enriched (Figure 3). All 5 functional annotations (‘cytoskeletal protein binding’, ‘sarcomere’, ‘myofibril’, ‘contractile fiber part’, and ‘actin cytoskeleton’) play a known role in the pathogenesis of CDI. The 7 genes linked to these functional annotations are shown in Supplementary Table 2 and include TNKS1BP1, JUP, TNNT1, SPTBN1, C22ORF28, and BCL2L11.

## DISCUSSION

Clinical manifestations of *Clostridium difficile* infection range from asymptomatic carriage to fulminant, pseudomembranous colitis and are mainly mediated by the effects of toxins A and/or B on intestinal epithelial cells. The expanding reservoir of *C. difficile* beyond the healthcare setting into community-based sources [3], and increasing use of NAAT tests to detect *C. difficile* and other gastrointestinal pathogens [27], has created challenges in accurately distinguishing CDI from other causes of diarrhea due to frequently overlapping clinical syndromes. When used in patients with known alternative causes of diarrhea (eg, laxative use) [28], a positive toxin result can lead to overestimation of disease and inappropriate treatment. Given the wide range of clinical manifestations of CDI, characterizing the host transcriptional response to infection has the potential to improve our understanding of the disease process and to identify expression profiles that correlate with outcome.

We demonstrate the potential use of host mRNA-targeted amplicon-based sequencing of fecal samples to identify gene transcripts enriched in patients with CDI compared to controls with *C. difficile-*negative diarrhea. We found upregulation of genes associated with cytokine responses previously shown to be important in *C. difficile* pathogenesis, including IL23A, which is associated with increased colonic inflammation and mortality in animal models of CDI [13, 29, 30], and IL34, which is upregulated in tissues of patients with inflammatory bowel disease and in mouse models of experimental colitis [31–33]. Intriguingly, we also found upregulation of genes related to actin-cytoskeleton function, including TNNT1, JUP, SPTBN1, and MYBPC3. The toxins of *C. difficile* act through disturbance of the intestinal cell cytoskeleton resulting in apoptosis [34, 35], and hypervirulent strains of *C. difficile* inhibit actin polymerization through production of an actin-ADP-ribosylating toxin [36]. Identification of such biologically plausible targets thus provides proof-of-concept for the use of host fecal transcriptomics to probe human mucosal immune responses.

Host transcript profiling has the potential to complement existing microbe-targeted molecular tests to improve specificity for detecting clinically relevant *C. difficile* infections. Existing tests have centered on microbial toxin detection in stools by functional assays (ie, cell cytotoxicity assay), detection of toxins A and/or B antigen, or NAATs for toxin genes (*tcdA* and/or *tcdB*). NAAT testing has improved our ability to rapidly detect genes for Toxins A and/or B, which represents a significant advance over detection of free toxin by antigen detection methods [37]. An accumulating body of evidence, however, questions the benefit of NAAT testing alone [38]. Specificity of NAAT decreases when clinical manifestations are taken into account [39]. Additionally, clinical outcomes in patients solely with PCR-positive results are similar to patients with negative results for *C. difficile* by both toxin assay and NAAT [40]. Updated *C. difficile* guidelines discourage testing with NAATs alone and recommend combining NAAT with toxin detection in multistep algorithms in conjunction with efforts to avoid inappropriate testing that might identify asymptomatic carriers of toxigenic *C. difficile* [41]. These recommendations were considered to be weak with low quality of evidence so new approaches are needed.

Host mRNA can be detected in feces by nucleic acid amplification tests and next-generation sequencing [20, 42–45]. However, host RNA contributes only a minute fraction to the total RNA present in fecal specimens with most of the total RNA being of bacterial origin (largely ribosomal RNA) [46]. Quantifying expression levels of host mRNA in this overwhelming background of bacterial RNA is technically challenging. In addition, the integrity of host cells and nucleic acid may be compromised. Next-generation sequencing technologies based on sequence-specific amplification of short regions of hundreds to thousands of mRNA transcripts (amplicon sequencing) provides a solution to this dilemma. Similar methods have been used for transcriptional profiling of RNA that is in low abundance or highly fragmented RNA, such as RNA extracted from formalin-fixed tissue [47, 48].

Our results provide proof-of-concept for the use of amplicon sequencing to noninvasively probe the mucosal host immune response to *C. difficile* infection. If confirmed in larger studies, this strategy will also enable noninvasive profiling and diagnosis of other gastrointestinal diseases such as other intestinal infections, inflammatory bowel disease, and colon cancer. In respiratory tract infections, a similar approach has led to the discovery of host mRNA expression signatures that can be used for diagnostic purposes and to differentiate bacterial from viral infections [49–52]. After defining a minimal set of maximally informative transcripts, quantitative reverse transcription PCR panels can be designed for faster, cheaper, and more scalable testing and development of tests that can be implemented in clinical laboratories [53]. While fecal samples provide unique technical challenges for nucleic acid extraction and detection, applications in noninvasive gastrointestinal cancer detection and multiplex panels for gastrointestinal pathogens have demonstrated that these can be overcome. Eliminating the need for colonoscopy and biopsy to assess the mucosal immune response provides a substantial benefit from our approach and an incentive to further optimize testing strategies.

Our study has several limitations. First, despite the large number of samples collected, only a small number of samples were analyzed by next-generation sequencing because of variable human mRNA yields (as measured by ACTG1:16S ratios), resource constraints, and the exploratory nature of this study. Despite the limited sample size, the biological plausibility of results (genes with a known role in pathogenesis were differentially expressed) demonstrates the power of this noninvasive approach. Second, our convenience sampling of stool specimens with > 4 mL and with higher levels of ACTG1 mRNA may have introduced a selection bias towards a certain phenotype of diarrhea caused by *C. difficile*. Future studies will include a carefully selected patient population with clinically confirmed *C. difficile* colitis, patients with diarrhea with a positive *C. difficile* test with an alternative cause of diarrhea identified, and randomly selected specimens to address this limitation. Third, further improvements in enriching human cells and/or RNA in stool specimens will improve sensitivity for expression analyses of less abundant genes and allow for a larger proportion of specimens to be analyzed. Lastly, we did not account for antibiotic use among patients, which may influence the host transcriptome. Despite these limitations, our study provides proof-of-concept that amplicon-based next generation sequencing of fecal samples can be used to probe host gene expression for immune profiling and biomarker discovery.

In summary, in our small, pilot study, we demonstrate differential expression of several genes in patients with *C. difficile* infection compared to those with diarrhea negative for *C. difficile*. Larger numbers of representative samples from patients with known *C. difficile* infection and diarrhea of other causes with *C. difficile* carriage will need to be tested to confirm and validate our findings and assess the power of host transcriptomics to assist in the critical distinction between *C. difficile* infection and carriage.

## Figures and Tables

**Supplementary Table 1.** Top 50 Genes Used for DAVID Analysis

UNKL	GPRC5C	WBP1L
FAM3A	NAALADL1	CDC26
AIP	HIPK2	FAM3C
PSMD11	MDK	SDSL
SNX4	POLD4	TARSL2
ARHGAP35	KCTD5	ALOX15
EXOSC8	NIPA1	TNNT1
SPTBN1	BCL2L11	TNKS1BP1
GTF2F1	EIF4A2	ARHGAP42
PLCB3	MPST	OTUD5
C22orf28	BMF	JUP
IFT122	SERPINB6	KLHDC8A
CDK8	EFNA3	DUSP23
MTA1	TARS	TUBB2A
RAP2A	SLC47A1	NFU1
SLC28A1	LZTS2	CHID1
NAA38	ANKRD11	

**Supplementary Table 2.** Enriched functional annotations of the top 50 differentially expressed genes (DAVID analysis) and included genes.

GO Term	GO Accession	Gene
Cytoskeletal protein binding	0008092	TNKS1BP1, JUP, TNNT1, SPTBN1, C22ORF28, BCL2L11
Sarcomere	0030017	JUP, TNNT1, SPTBN1
Myofibril	0030016	JUP, TNNT1, SPTBN1
Contractile fiber part	0044449	JUP, TNNT1, SPTBN1
Actin cytoskeleton	0015629	JUP, TNNT1, SPTBN1, BMF
